# Foreign Correspondence

**Published:** 1869-08

**Authors:** 


### Foreign Correspondence.

R. R. ALLGEMEINEN KRAUKENHAUSE,
VIENNA, *July 3d, 1869.*

DEAR EXAMINER:—Some time since I gave a somewhat detailed account in your columns of the opportunities and methods of studying Surgery, Obstetrics, etc., in Vienna. Most of the other branches of the science are studied in a somewhat similar manner, and it may be proper to give them a more cursory consideration.

The clinics on internal medicine are given by Professors Oppolzer and Skoda, in the wards at the bedside. It is much to be lamented that amphitheatres are not provided, and better opportunities given to students for hearing these wide-reputed men. Prof. Oppolzer has probably upwards of two hundred students at his clinics, about thirty of whom surround the patient, and are able to hear the learned man's lecture. A large part of the class, however, hear nothing, wander up and down the wards, talk with the patients, or each other, read newspapers, perhaps hear a word occasionally, or arrange themselves about the bed of the next probable patient. For a foreigner, with an imperfect knowledge of the language, it is hardly worth while to attend these clinics, unless he be a *Frau* doctor, to whom a front position is accorded, as is the case with a young lady from New York. Each patient's history is noted by some one of the students from day to day, who reports the case when called upon, and is often subjected to a close questioning on the whole scope of the disease. All the lectures I have heard were given in one male ward of about eighteen beds. I understand lectures are sometimes given in the female wards.

The assistants of these professors give private courses through the semester on laryngoscopy and rhynoscopy, percussion and auscultation. Their classes are generally limited to twelve, and are provided with abundant material.

These courses continue from four to six weeks, with a fee of $9.00 each.

Drs. Benedict, Fieber, and Rosenthal give courses on electricity, with explanations of the machines, and applications of the element to patients. They are thorough students of this branch of therapeutics, which plays an important role in the hospital. Dr. Benedict's book on the subject is one of the latest and best books published. Not enough attention is yet given to this branch in America.

Private courses on the ear are given by Drs. Gruber and Politzer, who have abundant anatomical preparations to illustrate their lectures, also a sufficient ambulatory. Students are supplied amply with mirrors and speculi, and allowed to examine patients and apply the air douche and other remedies. Gruber has an ambulatory of from ten to forty patients daily, and in many respects affords superior advantages to Politzer, whose ambulatory and supply of instruments are smaller. In most cases Gruber introduces the catheter into the eustachian tube, and so inflates the ear; while Politzer generally introduces a shorter tube simply into the cavity of the nose, and applies the douche. In other respects their treatment is pretty much the same. The respective claims of these two methods I have not space to consider. Dr. Mathewson, of Brooklyn, has been translating Politzer's Monogram on the Tympanum, I believe, which will probably soon be in the hands of the profession.

Professors Arlt and Ed. von Yäger have charge of the Eye Department. Each has from sixty to eighty beds, and a large number of out-patients. Candidates for graduation are only required to attend Arlt's clinic, hence Yäger is mostly followed by foreigners, as the clinic is not crowded. Concerning Arlt's clinic, I translate a few lines from a pamphlet, written and published by some of the students. "It is truly laughable to find the operation hall for ophthalmic knowledge a two-windowed room, in which stand six partly straight and partly half circular benches, which altogether, according to numbers, represent forty-nine places; when it is known that for these lectures, during the last semester, 130 to 150 students were inscribed. The old portraits on the wall show sufficiently that the room, in times gone by, was devoted to the eye clinic, times, however, in whicn the number of students of medicine did not reach 1058." I quote a few more words in a foot-note: "For a demonstration, since how long a time no reorganization has been made by the faculty, corresponding to the conditions of science, the following yet existing law (1869) may serve. It says: 'No professor of obstetrics shall undertake an operation in his clinic without first having obtained the approval of the head midwife.'"

Yäger's clinic is from 8 to 10, and Arlt's from 10 to 12 A.M., thus enabling those who make the eye an especial study to devote the whole forenoon to patients. About 8000 eye patients are treated yearly.

Their assistants give private courses on opthalmoscopy, refraction and accommodation operations, etc., and afford fine opportunities for gaining a knowledge of these topics.

Prof. Hebra's clinic for skin diseases is mostly attended by foreigners, who pay a double fee, $15.00. He treats about 4000 patients yearly. Male house patients are divested of their entire clothing, and pass about the class. Female patients are not required to expose the genitals.

Private courses are also given by his son-in-law, Dr. Köhn. Also by Newman, who has recently written a book on skin diseases, of some value. Also by one or two other men.

Prof. Sigmund gives three or more courses, of six weeks each, during the year, on venereal disease.

His assistant gives some private courses. Males and females are stood nude, on benches, to be examined by the class. The professor speaks very fast, and frequently considerable portions of his lectures are in Latin, so that the unclassical scholar, with also a poor knowledge of the German, learns little more than he sees.

Courses are given on pathological anatomy, for which the *post mortems*, averaging perhaps six per day, afford abundant material.

Other courses on microscopy, histology, etc., are given by the assistants of Rokitansky, who furnish instruments and other apparatus.

There are also courses given on anatomy and pathology of the brain, insanity at the asylum, urinalysis in the laboratory, which are said to be good, but I have not been able to take them, or visit them. Rokitansky lectures daily; but few Americans go to hear him.

Many of the hospital wards are miserably ventilated, and crowded. There is only one general bath-room for the whole hospital. Numbers of patients lie in their beds for weeks in a state of uncleanliness and almost filth, inconsistent with health or decency. In some of the wards they are disturbed at all hours of the day by private courses, and scarcely procure necessary rest. Many of them are required to sacrifice too much for the student. Some of the students seem to think patients are simply here for their benefit, and treat them accordingly.

Students beginning the study of medicine had better do so at home, or elsewhere than here, as the conveniences for such study are not at all equal to the number of students; *e. g.*, nearly two hundred of the students of physiology are obliged to stand during the lectures. The arrangements for anatomy and chemistry, I understand, about the same.

Hastily, F.

